# Hemodynamic and Morpho-Biochemical Parameters of Rabbit Blood After Injection of Enzyme Preparations

**DOI:** 10.3390/biom15071049

**Published:** 2025-07-18

**Authors:** V. G. Vertiprakhov, N. A. Sergeenkova, S. V. Karamushkina, B. Sh. Dashieva

**Affiliations:** Department of Physiology, Ethology and Biochemistry of Animals, Russian State Agrarian University—Moscow Timiryazev Agricultural Academy, 49, Timiryazevskaya St., Moscow 127550, Russia; nsergeenkova@rgau-msha.ru (N.A.S.); sveta.vetmed@mail.ru (S.V.K.)

**Keywords:** trypsin crystalline, lyophilizate pancreas pigs, rabbits, blood pressure, blood trypsin

## Abstract

The anti-inflammatory effect of trypsin in animals and humans is the basis for the development of new veterinary and medical drugs and alternatives to antibiotics. The current experiment analyzed the effect of pig pancreatic tissue lyophilizate and crystalline trypsin on the hemodynamic and morpho-biochemical parameters of rabbit blood. The experiments were carried out on 20 rabbits of the Soviet chinchilla breed of 6–8 months of age. Animals were intramuscularly injected with sterile solution of 0.9% NaCl in 0.5 mL (group 1, n = 5), sterile solution of crystalline trypsin in 0.9% NaCl at a concentration of 0.25 mg/kg body weight (group 2, n = 5), sterile solution of crystalline trypsin in 0, 9% NaCl at a concentration of 0.5 mg/kg body weight (group 3, n = 5), or sterile suspension of pig pancreas lyophilizate at a concentration of 1 mg/kg body weight (group 4, n = 5). Animals were injected once daily for five consecutive days. Significant changes in arterial blood pressure, serum enzymes activity, and the count of various blood cellular components were induced by the administration of different trypsin preparations. All data obtained indicate the presence of a biologically active substance in the lyophilizate, the effect of which requires further animal studies to create a prototype for the development of new drugs for human and animal use.

## 1. Introduction

The problem with finding efficient alternatives to antibiotics is extremely relevant today. Trypsin is known to have anti-inflammatory and decongestant effects [1,2,3], which in its effectiveness can be compared to nonsteroidal, anti-inflammatory drugs. The use of enzyme preparations from animal tissue, including those prepared from the pancreas, has limited use in animal husbandry practice, since the crystalline trypsin is mainly used to treat wounds and inflammatory processes. The relevance of studying pancreatic proteases, secreted by digestive glands into the blood, is important due to the large number of studies performed in recent years on receptors activated by proteases. Receptors are located on the cell membranes of various organs and tissues, through which the functional activity of these organs and tissues can be enhanced or reduced under the influence of pancreatic proteases [4,5]. Trypsin has been found to be one of the activators of PAR receptors [6]. A study by Koshikawa N., Hasegawa S., Nagashima Y. et al. (1998) [7] showed trypsin expression in human and mouse non-pancreatic tissues. The trypsin gene was found to be expressed at high levels in the pancreas, spleen, and small intestine. In situ hybridization and immunohistochemistry have shown that trypsin is widely expressed in the epithelial cells of the skin, esophagus, stomach, small intestine, lungs, kidneys, liver, and extrahepatic bile ducts, as well as in the spleen and neurons [7]. Thus, the enzyme trypsin is often considered to be a hormone-like substance [8], which, when entering the blood, can have a regulatory effect on the behavior of animals [9], changes in blood pressure and heart rate in rabbits [10], as well as on metabolism in general [11].

The production of enzyme preparations for parenteral use depends not only on the technology, but also on the raw materials from which the enzyme is isolated. The pharmacopeial preparation, trypsin crystalline, is obtained from the pancreas of cattle. There is evidence that the proteolytic activity in the homogenate of the pancreas of cattle is 405 ± 4.9 mg casein/g pancreas tissue/min [10]. Protease activity reaches 839 ± 87.4 mg/g/min in the pancreatic tissue of broiler chickens and 317 ± 13.9 mg casein/g pancreas tissue/min in that of pigs. Differences in the activity of biological raw materials indicate the possibility of studying different options for the preparation of enzyme preparations. A previous study from our lab showed that an injectable drug (lyophilizate from poultry pancreatic tissue) contributed to an increase in the blood pressure of rabbits [8]. The aim of the current study was to determine the effect of crystalline trypsin and lyophilizate from the pancreatic tissue of pigs on the hemodynamic parameters and morpho-biochemical status of rabbits. The current study is also the first study that presents the results of testing a new lyophilic drug prepared from the pancreas of pigs.

## 2. Materials and Methods

### 2.1. Animals and Care

The experiments were carried out on rabbits of the Soviet chinchilla breed, age 4.0–6.0 months, body weight of at least 3.5 kg. They were fed with complete granulated feed for rabbits (GOST 32897-2014), in an amount of 100–110 g per day, administered twice per day.

The feed comprised wheat bran, sunflower meal, alfalfa grass meal, barley, wheat, limestone meal, vegetable oil, and a vitamin and mineral premix (crude protein—18.50%, crude fiber—11.13%, metabolizable energy—207.00 Kcal/100 g). Ethical approval was obtained from the Commission on Bioethics of the Russian State Agrarian University—Moscow Timiryazev Agricultural Academy (extract from the protocol application for research using animals No3 from the 7 April 2023 (07/04/02023)). Rabbits in the groups were selected according to the principle of analogs.

The experiment was carried out using the group–period method; the scheme is presented in Table 1.

### 2.2. Tested Substances and Their Preparation

Crystalline trypsin (Samson-Med LLC, St. Petersburg, Russia) contains 10 mg of active trypsin per vial, obtained from the pancreas of cattle. Before use, the drug was diluted with saline at a rate of 5.0 mL per vial and injected intramuscularly into the pelvic limb of the rabbit, at a dose of 0.25 mg/kg body weight. When the dose of crystalline trypsin was increased 2-fold, the amount of 0.9% sodium chloride solution was reduced to 2.5 mL and 0.5 mL was injected intramuscularly. Lyophilizate from the pancreas of pigs was prepared using a special technology developed in the scientific laboratory of physiology of animal nutrition of the Russian State Agrarian University—Moscow Timiryazev Agricultural Academy using a lyophilic dryer AK 6–50 (LioGene, Russian Federation). After freezing (−20 °C), the pancreata were placed in vials and dried in a freeze-dryer with the preservation of their properties at the following parameters: temperature −53 °C, pressure 0.015 MPa for 48 h. After drying, the lyophilized powder was packaged in vials of 50 mg and sterilized in an autoclave with saturated steam at 0.5 atm, 114 °C for 30 min. The vials were then sealed with sterile rubber stoppers and aluminum caps. Determination of enzyme activity using the previously described biochemical method [7], using the substrate N-a-Benzoyl-DL-Arginine-4-Nitroanilide Hydrochloride (L-BAPNA) (Sisco Research Laboratories Pvt. Ltd., India), showed that the lyophilized powder has a proteolytic activity of 1768 ± 110.5 U/L [6]. The results of studies to determine trypsin activity in a preparation from the pancreatic tissue of pigs showed that the activity was 432 ± 39.3 U/L. Since the activity of the drugs differed, the dose of lyophilizate chosen for the current study was 1.0 mg/kg of body weight of the rabbits. The drug was diluted before use with 6.25 mL of 0.9% sodium chloride solution and injected suspension intramuscularly into the pelvic limb of the rabbits at 0.5 mL.

### 2.3. Blood Pressure and Heart Rate Measurements

**Figure 1:**
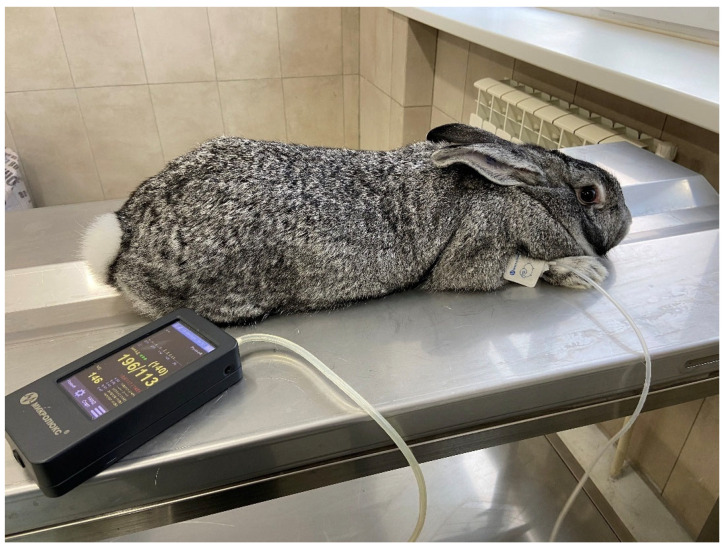
Measuring blood pressure and heart rate in a rabbit.

The procedure for determining the blood pressure (BP) and heart rate in the rabbits was performed daily in the morning hours, before feeding, using an automatic veterinary tonometer—ML-430 VET (Microlux, RF) (Figure 1). To perform this, the rabbits were fixed on a table and the cuff was placed on the front leg of the rabbit and blood pressure was measured at least five times in a row. The device recorded systolic, diastolic, and average blood pressure, as well as heart rate (HR). After injection of the drug, the procedure for measuring blood pressure and heart rate was repeated.

### 2.4. Blood Sampling

Blood for biochemical studies in rabbits was obtained from the ear vein into test tubes with a coagulation activator containing a silicon oxide (SiO_2_) filler. Blood sampling was performed in the morning in a fasted state, after completion of the series of experiments (after 5–7 days). Serum was separated and frozen at −20 °C for further analysis.

### 2.5. Blood Biochemical Analysis

Blood biochemical analysis (amylase activity, total protein, glucose, triglycerides, cholesterol, alkaline phosphatase, uric acid, calcium, and phosphorus) was performed on an automatic biochemical analyzer—BioChem FC-120 (High Technology, Inc., North Attleboro, MA, USA), using reagent kits from this company. Trypsin activity was determined using a biochemical method on a BS-3000M analyzer (Sinnowa, China), using the substrate N-benzoyl-DL-arginine-n-n-nitroanilide (BAPNA) [7]. For this purpose, 450 µL of buffer solution (pH 8.2)—Reagent 1—was placed into an Eppendorf tube and 50 µL of Reagent 2, containing the trypsin substrate, was then added. Reagent 2 was prepared as follows: 5.0 mg of N-benzoyl-DL-arginine-n-n-nitroanilide (BAPNA) powder was dissolved in 1.0 mL of dimethyl sulfoxide, stirring constantly for 2–3 min. The solution can be stored in a refrigerator at +4 °C for up to 3 months. For the analysis, a mixture of Reagents 1 and 2 was stirred in a closed Eppendorf tube and warmed for 3 min in the thermostat at +37 °C. After incubation, 10.0 µL of the test material (serum) was added and mixed with the reagent mixture and the reaction on the biochemical analyzer was started. The mode for enzyme determination was pre-set on the device: main filter—405 nm, delay—15 s, measurement time—60 s, blank sample—water, factor—4554; calibration was performed using a TruCal multicalibrator (Di Sys Diagnostic Systems GmbH, Germany). The unit of trypsin activity measurement was U/L.

Blood morphological parameters were determined on an automatic hematological analyzer—MicroCC (MicroCC20Plus, MCC-2002-VO-RU, High Technology, Inc., USA).

### 2.6. Statistical Analysis

STATISTICA 14.0.0.15 (TIBCO Software Inc.) was used for statistical analyses. Data were tested for normal (Gaussian) distribution using the Kolmogorov–Smirnov test, Lilliefors test, or Shapiro–Wilk normality test depending on the sample size. Within-group between-period comparisons were made using either a paired t-test or a Wilcoxon test, depending on the distribution of the data. Differences in investigated parameters between experimental groups were assessed using a one-way ANOVA for normally distributed datasets and a Kruskal–Wallis test for data with non-Gaussian distribution. Homogeneity of variances in the groups was checked with the Levene test. Independent samples with unequal variances were assessed using Welch’s ANOVA test. Differences were considered significant if *p* < 0.05 and *p* < 0.1 was considered as a trend.

## 3. Research Results

### 3.1. Blood Pressure and Heart Rate

The effects of the pancreatic and crystalline trypsin preparation on the blood pressure and heart rate of rabbits are presented in Table 2.

In the control group (group 1), no significant changes in blood pressure indices were observed after the injection of 0.9% sodium chloride solution. Table 2 shows that after the administration of crystalline trypsin to rabbits at a dose of 0.25 mg/kg body weight, systolic blood pressure decreased by 11.4% (*p* < 0.05), diastolic blood pressure by 11.5% (*p* < 0.05), mean blood pressure by 11.2% (*p* < 0.05), and heart rate by 7.1% (*p* < 0.05) compared to background.

Interestingly, a 2-fold increase in the dose of crystalline trypsin (group 3) did not result in any changes compared to baseline. At the same time, the injection of pig pancreas lyophilizate (group 4) resulted in a significant decrease in systolic pressure (by 8.4%, *p* < 0.05); diastolic pressure (by 7.7%, *p* < 0.05); and mean arterial pressure (by 8.8%, *p* < 0.05); while heart rate increased by 5.0% (*p* < 0.05). Comparative analysis of the post-injection periods in different groups showed that in group 3, systolic, diastolic, and mean arterial pressure exceeded the values observed in the rabbits of group 2 (*p* < 0.05).

### 3.2. Blood Biochemical Indices

Blood biochemical indices reflect the metabolic processes and animals’ health status. In the current study we determined the activity of digestive enzymes in blood serum to assess the adaptation of the rabbits to the action of the test substances. The results are presented in Table 3.

The intramuscular administration of enzyme preparations had an inhibitory effect on the trypsin activity in the blood of rabbits, which decreased by 51.9% (*p* < 0.05) in the group 3 rabbits compared to the control group (group 1). At the same time, the highest dose of trypsin administered intramuscularly resulted in a significant increase in serum amylase activity by 16.9% (*p* < 0.05) compared to group 1.

An interesting result was obtained with respect to the alkaline phosphatase activity in the serum. This index decreased by 46.4% (*p* < 0.05) in group 2 after the administration of crystalline trypsin at a dose of 0.25 mg/kg body weight, by 43.5% (*p* < 0.05) in group 3 after the administration of crystalline trypsin at a dose of 0.5 mg/kg body weight, but the administration of pig pancreas lyophilizate (group 4) led to a significant increase in alkaline phosphatase activity by 2.2 times (*p* < 0.05) compared to group 1. The level of total protein in the serum also increased slightly in the animals of group 3 (by 7.6%, *p* < 0.05) compared to group 1.

The serum uric acid levels were significantly reduced in group 3 (*p* < 0.05) compared to the rabbits of group 1. The serum phosphorus level was significantly decreased in the rabbits of group 2 and 3 compared to the rabbits of group 1 (*p* < 0.05).

Morphological blood indices reflect the cellular metabolism and the state of the body’s immune system. The results of determining the content of erythrocytes, leukocytes, and platelets in the blood of rabbits are presented in Table 4.

The morphological parameters of the blood of experimental group 2, the rabbits of which were administered crystalline trypsin in the minimum dose (0.25 mg/kg live weight), had no significant differences from control group 1. The number of leukocytes after the administration of the highest dose of crystalline trypsin (group 3) decreased by 29.4% (*p* < 0.05) compared to group 1 and reached the physiological minimum. The administration of pig pancreas lyophilizate did not affect the leukocyte count (group 4). Lymphopenia was observed in control group 1 and experimental groups 2 and 3. Interestingly, the administration of pig pancreas tissue lyophilizate increased the number of lymphocytes by 64.6%, up to the normal reference range. In contrast, the number of granulocytes in the blood of the rabbits of groups 2 and 3 after the administration of enzyme preparations was at the level of group 1, with group 2 being higher than group 4 by 44.0% (*p* < 0.05). At the same time, the ratio of granulocytes to lymphocytes between group 1 and group 4 changed by 1.8 times.

## 4. Discussion

The current study assessed the effects of various enzyme preparations on the blood pressure, heart rate, and morpho-biochemical blood indices of rabbits. The blood pressure of rabbits has been shown to vary greatly, which becomes relevant when using modern blood pressure measurement devices. Systolic blood pressure in rabbits between the ages of 6 and 8 months has been shown to be 183.5 ± 12 mmHg, whereas diastolic pressure is 125.5 ± 7.5 mmHg. It is known [12,13] that when determining blood pressure in rabbits using an invasive method, the index was 74–100 mmHg. It should be noted that measurements performed by invasive and noninvasive methods may differ.

Enzymes transported into the bloodstream are known to form a pool of deposited zymogenic and active pancreatic enzymes adsorbed by plasma proteins and the endothelium of blood capillaries [14]. These enzymes are desorbed under certain conditions and are included in the pool of enzymes recreated by glandulocytes, together with the pool of enzymes synthesized at the same time by acinocytes. This explains the fact that the amount of pancreatic enzymes transported to the duodenum is substantially greater than the amount that can be synthesized at the maximum of pancreatic secretion, stimulated by food intake or other pathways [15,16,17]. In our experiments on rabbits, the effects of the enzyme preparations are most probably ‘realized’ within the nervous system, in the form of a change in blood pressure, during 30–60 min following administration. Previous studies have shown that crystalline trypsin injected into rabbits intramuscularly has an effect on the hemodynamics, heart rate, and morpho-biochemical parameters of the blood. Data presented here indicate that the crystalline trypsin at a dose of 0.25 mg/kg body weight and lyophilizate from the pancreatic glands of pigs at a dose of 1.0 mg/kg body weight had the same effect on the blood pressure of rabbits. Crystalline trypsin at a dose of 0.5 mg/kg body weight had no significant effect on the hemodynamics; apparently, the above dose exceeded the optimal value. Consequently, preparations from the porcine pancreas have an effect on the hemodynamics of rabbits at a certain dose, which suggests the effect of enzymes during parenteral administration on the metabolism of animals. One can assume that the adaptation of rabbits to the action of the enzyme preparations during the five days of the experiment varies depending on the dose and quality of the preparation.

Following the intramuscular injection of trypsin, the trypsin enters the blood and probably inhibits the existing serum trypsin, causing a reaction aimed at changing the metabolism within the whole body. To study the mechanism of action of serum trypsin on animal metabolism, an experiment was conducted to increase the dose of the intramuscular injection of crystalline trypsin 2-fold compared to that used previously. With an increase in the dose of intramuscularly injected trypsin, a significant dose-dependent decrease in serum trypsin activity was noted. For the moment, one thing is obvious—the trypsin preparations increased the influence of the parasympathetic part of the nervous system, which has a positive effect on the assimilation of nutrients and anabolic processes in tissues.

Trypsin also affects hemodynamics through the parasympathetic nerves [18], since the main mechanism of nervous regulation of blood pressure and heart rate is the baroreflex [19], which differs from humoral mechanisms in the speed of reaction. The sensory nerve endings of the depressor nerve, which is a branch of the vagus nerve, are located in the aortic arch. Nerve impulses coming from receptors along the depressor and vagus nerves to the medulla oblongata cause a decrease in the activity of neurons in the pressor zone of the vasomotor center, which leads to an increase in the lumen of blood vessels and a decrease in blood pressure [20,21,22,23,24]. The results of the studies indicate a change in some indicators in the biochemistry of the blood. A decrease in serum trypsin activity with parenteral enzyme preparations may be associated with the regulation of pancreatic secretory function by the feedback principle [25]. It has been reported that a similar response is observed in pancreonecrosis, where exogenous trypsin inhibits trypsin secretion, probably by a short negative feedback mechanism, namely the trypsin–acinar cell axis [26]. The concept of feedback in the regulation of pancreatic secretion emerged as a result of a series of studies demonstrating that the injection of trypsin inhibitor into the upper part of the small intestine or surgical clamping of the bile duct removing bile and pancreatic juice from the duodenum of rats stimulated the secretion of pancreatic enzymes [27,28,29]. However, the regulation of pancreatic regulation and metabolism by pancreatic enzymes in serum remains poorly understood, and circulating enzyme data are sparse [30]. To date, the mode of entry of pancreatic enzymes into the blood and pancreas remains open to debate [31].

The intramuscular injection of enzyme preparations led to the inhibition of serum trypsin activity. This indicates the competitive effect of trypsin in the blood after intramuscular injection. Changes in trypsin activity in the blood may be due to the fact that trypsin provides a dynamic equilibrium between the activity of proteolytic and inhibitor systems in the process of hemostasis [32] and accelerates the processes of lipid peroxidation (LPO), which is explained by the proteolytic effect of trypsin [33]. An increase in serum trypsin levels is observed in pancreatic inflammation [34,35]. Consequently, despite the data on the effect of trypsin on the regulation of enzyme secretion in the pancreas, the issue of trypsin correlation in the intestine and blood remains poorly studied and requires further research, since the role of trypsin is not limited to protein hydrolysis in the intestine, but goes far beyond this process.

The change in the blood biochemical parameters in the rabbits in group 2 of the current study indicates a change in the metabolism associated with the action of trypsin. The decrease in the trypsin activity in the blood of rabbits of the 3rd and 4th groups, apparently, is associated with the metabolism and adaptation to the intake into the body during the parenteral administration of exogenous trypsin in an increased dose. As the hemodynamic and blood biochemistry parameters show, the application of crystalline trypsin has a more pronounced effect on the organism of rabbits in comparison with the lyophilizate from pig pancreatic gland tissue.

This leads to a change in the ratio of granulocytes to lymphocytes from 2.43 to 1.03, which indicates an increase in the function of the immune system responsible for the specific defense of the body. The number of erythrocytes, which perform the main function of oxygen transport, did not change after the administration of the enzyme preparations.

The ratio of lymphocytes to heterophils in the blood is associated with the state of the immune system, as well as the stress response [36]. It has been established [37] that a change in the average volume of platelets follows a change in the rate of their production. The link between megakaryocyte size and ploidy suggests that lymphoma increases the DNA content of these cells. Therefore, the data obtained in the current study on the effects of porcine pancreatic lyophilizate and crystalline trypsin on the body of rabbits are new, allowing us to expand the idea of the possibilities of using trypsin as part of complex therapy for circulatory pathology, digestion, and metabolic disorders.

## 5. Conclusions

The results of the present study of the effect of various preparations containing enzymes of bovine pancreas (crystalline trypsin) and pig pancreas (lyophilizate) showed that after the administration of crystalline trypsin, the mean arterial pressure and heart rate in rabbits decreased by 11.2% (*p* < 0.05), while the administration of pig pancreas lyophilizate decreased the mean arterial pressure by 8.8% (*p* < 0.05). Increasing the dose of crystalline trypsin by 2 times did not lead to a significant change in blood pressure, which indicates that the optimal dose of the drug was exceeded and that there is a regulatory mechanism in the parenteral administration of pancreatic preparations. At the same time, carbohydrate, fat and protein metabolism changed, the number of lymphocytes and the activity of enzymes such as trypsin, amylase and alkaline phosphatase increased, compared to group 1, which was administered with 0.9% NaCl solution. All this indicates the presence of a biologically active substance in the lyophilizate, the effect of which requires further animal studies to create a prototype for the development of new veterinary and medical drugs.

## Figures and Tables

**Table 1:** Scheme of the experiment.

Groups			
Group 1 control, n = 5 Injection of intramuscular saline 0.5 mL/head	Group 2, n = 5 Intramuscular injection of trypsin crystalline solution in 0.9% NaCl 0.25 mg/kg body weight	Group 3, n = 5 Intramuscular injection of trypsin crystalline solution in 0.9% NaCl, 0.5 mg/kg body weight	Group 4, n = 5 Intramuscular injection of pig pancreatic lyophilizate 1.0 mg/kg body weight
Period	1 Indicators of hemodynamics and heart rate before injection of the solution		
	2 Hemodynamic and heart rate parameters 60 min after injection of the drug		

**Table 2:** Blood pressure and heart rate in rabbits after intramuscular injections of different enzyme preparations.

Parameter	Group							
	1		2		3		4	
Period	Baseline	Post-injection	Baseline	Post-injection	Baseline	Post-injection	Baseline	Post-injection
Systolic pressure, mmHg	161 ± 3.9	1 62 ± 4.3	167 ± 27.7	148 ± 23.8 *^ab^	162 ± 32.3	159 ± 31.6 ^b^	166 ± 32.5	152 ± 30.5 *^a^
Diastolic pressure, mmHg	100 ± 3. 0	99 ± 3.7	104 ± 16.2	92 ± 15.4 *^ab^	101 ± 20.5	99 ± 20.5 ^b^	104 ± 22.4	96 ± 20.6 *
Mean pressure, mmHg	12 0 ± 2.6	12 0 ± 3.1	125 ± 19.6	111 ± 17.7 *^ab^	121 ± 24.6	119 ± 23.9 ^b^	125 ± 25.5	114 ± 23.4 *
Heart rate, bpm	215 ± 20.1	212 ± 20.1	226 ± 31.5	210 ± 37.5 *	215 ± 43.2	212 ± 40.4	202 ± 39.4	212 ± 39.9 *

* within-group between-period difference significant at *p* < 0.05; ^a^—difference with the control group, ᵇ—difference between group 2 and group 3 significant at *p* < 0.05. Data are presented as mean ± SD.

**Table 3:** Blood biochemical parameters of rabbits after intramuscular injections of different enzyme preparations.

Indicator	Group			
	1	2	3	4
Trypsin activity, U/L	79 ± 34.3	68 ± 2.3	38 ± 12.7 ^a^	47 ± 9.0
Amylase activity, U/L	154 ± 2.5	156 ± 8.6 ^b^	180 ± 7.6 ^abd^	157 ± 7.3 ^d^
Alkaline phosphatase activity, U/L	69 ± 3.7	37 ± 3.7 ^ac^	39 ± 10.8 ^ad^	153 ± 4.3 ^acd^
Total protein, g/L	66 ± 1.7	65 ± 1.8 ^b^	71 ± 2.8 ^abd^	65 ± 1.6 ^bd^
Uric acid, mmol/L	44 ± 2.6	44 ± 2.0 ^b^	22 ± 3.0 ^abd^	39 ± 7.2 ^d^
Glucose, mmol/L	6.3 ± 0.56	6.0 ± 0.54 ^c^	6.2 ± 0.74 ^d^	7.9 ± 1.23 ^acd^
Triglycerides, mmol/L	0.6 ± 0.06	0.6 ± 0.11	0.6 ± 0.06	0.6 ± 0.04
Cholesterol, mmol/L	2.7 ± 0.59	1.0 ± 0.04 ^a^	1.0 ± 0.00 ^a^	1.0 ± 0.00 ^a^
Calcium, mmol/L	3.3 ± 0.16	3.3 ± 0.04	3.6 ± 0.21	3.4 ± 0.16
Phosphorus, mmol/L	2.9 ± 0.29	1.5 ± 0.05 ^abc^	0.9 ± 0.16 ^abd^	3.1 ± 0.33 ^cd^

^a^—difference with the control group, ᵇ—difference between group 2 and group 3, ^c^—difference between group 2 and group 4, ^d^—difference between group 3 and group 4 significant at *p* < 0.05. Data are presented as mean ± SD.

**Table 4:** Morphological parameters of rabbits during intramuscular injection of different enzyme preparations.

Parameter	Group			
	1	2	3	4
WBC, ×10^9^/L	6.8 ± 0.46	7.3 ± 0.58 ^b^	4.8 ± 0.96 ^abd^	7.6 ± 1.40 ^d^
LYM, %	28.8 ± 2.17	29.0 ± 2.24 ^c^	29.5 ± 19.92 ^d^	47.4 ± 6.31 ^cd^
GRA, %	65.9 ± 3.37	69.1 ± 2.10 ^c^	64.6 ± 18.22	48.0 ± 2.92 ^ac^
Ratio of granulocytes to lymphocytes	2.30 ± 0.26	2.40 ± 0.26 ^c^	3.2 ± 1.97	1.03 ± 0.19 ^ac^
RBC, ×10^12^/L	5.9 ± 0.13	5.9 ± 0.12	6.7 ± 1.47	5.7 ± 0.37
HGB, g/L	135 ± 4.0	137 ± 5.2	133 ± 13.6	142 ± 8.6
MCHC, g/L	350 ± 17.7	343 ± 5.4 ^bc^	359 ± 7.4 ^b^	376 ± 12.9 ^ac^
HCT, %	35.9 ± 3.54	35.9 ± 3.54	41.7 ± 9.88	36.6 ± 0.68
PLT, ×10^9^/L	491 ± 65.9	558 ± 19.2 ^b^	430 ± 12.0 ^bd^	501 ± 30.0 ^d^
P-LCR,%	8.4 ± 0.69	8.1 ± 0.58 ^bc^	12.0 ± 2.08 ^ab^	13.5 ± 2.83 ^ac^

^a^—difference with the control group, ᵇ—difference between group 2 and group 3, ^c^—difference between group 2 and group 4, ^d^—difference between group 3 and group 4 significant at *p* < 0.05. Data are presented as mean ± SD. WBC—leukocytes count; LYM—lymphocytes count; GRA—granulocytes count; RBC—red blood cells count; HGB—hemoglobin; MCHC—mean corpuscular hemoglobin concentration; HCT—hematocrit; PLT—platelets count; P-LCR—platelets/large cells ratio.

## Data Availability

The original contributions presented in this study are included in the article. Further inquiries can be directed to the corresponding authors.
